# To construct a ceRNA regulatory network as prognostic biomarkers for bladder cancer

**DOI:** 10.1111/jcmm.15193

**Published:** 2020-03-31

**Authors:** Jiazhi Jiang, Yaqiong Bi, Xiao‐Ping Liu, Donghu Yu, Xin Yan, Jie Yao, Tongzu Liu, Sheng Li

**Affiliations:** ^1^ Department of Biological Repositories Zhongnan Hospital of Wuhan University Wuhan China; ^2^ Department of Urology Zhongnan Hospital of Wuhan University Wuhan China; ^3^ Tumor Precision Diagnosis and Treatment Technology and Translational Medicine Hubei Engineering Research Center Wuhan China

**Keywords:** bladder cancer, ceRNA, co‐expression network, prognostic marker

## Abstract

Emerging evidence demonstrates that competing endogenous RNA (ceRNA) hypothesis has played a role in molecular biological mechanisms of cancer occurrence and development. But the effect of ceRNA network in bladder cancer (BC), especially lncRNA‐miRNA‐mRNA regulatory network of BC, was not completely expounded. By means of The Cancer Genome Atlas (TCGA) database, we compared the expression of RNA sequencing (RNA‐Seq) data between 19 normal bladder tissue and 414 primary bladder tumours. Then, weighted gene co‐expression network analysis (WGCNA) was conducted to analyse the correlation between two sets of genes with traits. Interactions between miRNAs, lncRNAs and target mRNAs were predicted by MiRcode, miRDB, starBase, miRTarBase and TargetScan. Next, by univariate Cox regression and LASSO regression analysis, the 86 mRNAs obtained by prediction were used to construct a prognostic model which contained 4 mRNAs (ACTC1 + FAM129A + OSBPL10 + EPHA2). Then, by the 4 mRNAs in the prognostic model, a ceRNA regulatory network with 48 lncRNAs, 14 miRNAs and 4 mRNAs was constructed. To sum up, the ceRNA network can further explore gene regulation and predict the prognosis of BC patients.

## 1 INTRODUCTION

Bladder cancer (BC) is among the leading cancers worldwide. For 30 years, the prognosis and treatment for BC had not made great progress.^1^, ^2^ The poor prognosis and the difficulty in treatment are bound to the alteration and regulation of genes. An alteration in genes that play a key role in cell cycle regulation can contribute to high‐grade tumours and may alter the sensitivity to drugs in BC.^3^ Recent studies demonstrate that the regulation of ceRNA network plays a crucial role in the occurrence and development of cancers.^4^, ^5^

The ceRNA hypothesis postulates that in addition to the conventional function, miRNAs targeting RNAs, a reversed logic exists. Any RNA transcript with miRNA response elements (MREs) could bind to miRNA, thereby regulating the expression of RNAs with the same MREs. Significantly, because the two transcripts share the same MREs, we can predict the ability of two transcripts to regulate each other based on MRE overlap.^6^, ^7^ There is increasing evidence that lncRNA, miRNA and mRNA are involved in the biological processes of cancer. Among the ceRNA, lncRNAs and mRNAs might harbour the same MREs. Therefore, when miRNA binds to MRE on lncRNA, mRNA expression is not inhibited which may contribute to disease pathogenesis.^8^ Currently, the ceRNA network has been shown to play an important role in the occurrence and progression of different types of cancers. But the roles of ceRNA network in BC, especially the analysis of ceRNA regulatory network based on large sample size and next‐generation sequencing, were not completely expounded. So, this research aims to construct a ceRNA regulatory network in BC.

## 2 METHODS

### 2.1 RNA‐Seq data and clinical characteristics of patients from TCGA

**Figure 1:**
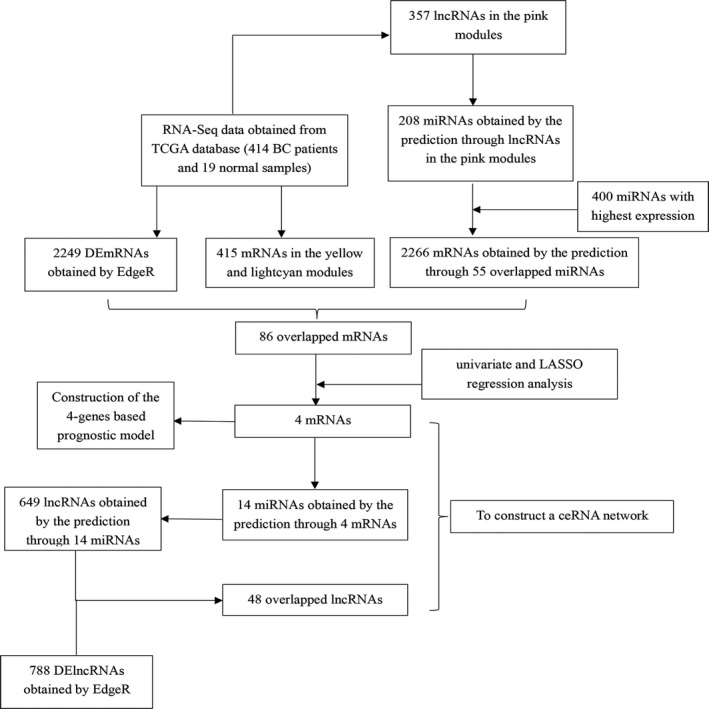
The flow diagram of this study

Figure 1 shows the flow diagram of our study. We obtained RNA‐Seq data of BC from the TCGA database (https://portal.gdc.cancer.gov/), which contained 414 bladder urothelial carcinoma samples and 19 normal bladder samples. Besides, TCGA also included the corresponding clinical characteristics (406 bladder urothelial carcinoma patients) including overall survival and other clinicopathological characteristics of BC patients.

### 2.2 Identification of differentially expressed mRNAs and lncRNAs

We transformed the ensemble IDs of BC samples into gene symbols based on GENCODE (https://www.gencodegenes.org/human/).^9^ We identified differentially expressed mRNAs (DEmRNAs) and differentially expressed lncRNAs (DElncRNAs) between bladder urothelial carcinoma and normal bladder tissue by means of EdgeR R package.^10^ And when they met these criteria (Adjust *P* < .05, and |log2 FC| ≥ 1), we considered mRNAs and lncRNAs as DEmRNAs and DElncRNAs.

### 2.3 Analysis of gene function and pathway enrichment

We performed Gene Ontology (GO)^11^ and Kyoto Encyclopedia of genes and Genomes (KEGG)^12^ and Gene Set Enrichment Analysis (GSEA)^13^ for DEmRNAs by means of clusterProfiler R package.^14^ We take advantage of GO to describe gene functions, including three aspects: biological process (BP), molecular function (MF) and cellular component (CC). KEGG‐GSEA was used to get possible pathways, and the cut‐off criteria for gene sets were set at *P* < .05.

### 2.4 Construction of a co‐expression network

WGCNA R package^15^ was conducted to construct a co‐expression network. First, unqualified genes were removed by goodSamplesgenes. Then, construct a sample network based on squared Euclidean distance, and samples with Z.k < −2.5 which were considered as outlying samples were excluded from subsequent studies. Then, the variances of mRNAs and lncRNAs across the left samples were calculated, and we chose the top 60% mRNAs and lncRNAs with highest variances for WGCNA. Next based on the scale‐free topology criterion, we chose an appropriate soft‐thresholding power (β) by pickSoftThreshold to set up a weighted adjacency matrix (WAM). Given the function of the topological overlap matrix (TOM),^16^ we transformed adjacency into TOM. After calculating module eigengenes, densely correlated and highly co‐expressed genes with BC patients are grouped into the same module.

### 2.5 Construction of the ceRNA network

LncRNAs in the key modules most relevant with BC were picked to predict miRNAs by the means of miRcode (http://www.mircode.org). Then, we explore target mRNAs of miRNAs by the MiRDB (http://www.mirdb.org/), miRTarBase (http://mirtarbase.mbc.nctu.edu.tw//), StarBase (http://starbase.sysu.edu.cn/) and TargetScan (http://www.targetscan.org//). Besides, Cytoscape software (https://cytoscape.org/) was used to visualize the relationship of ceRNA network.

### 2.6 Construction of BC prognostic signatures

We collected the clinical data of 186 BC patients with both OS and DFS. At the same time, for verification, we randomly divided patients into two groups based on the ratio of 5:4 (the training set = 103 and internal test set = 83). We conducted the chi‐square test to analyse the differences in clinical features including age, gender, tumour subtype, differentiation grade, T stage, TNM stage and survival status between training set and internal testing set(the N stage and M stage are discarded because of patients with DFS data only being N0 or M0 status). By means of the ‘survival’ package in R, univariate Cox regression method was conducted to explore the correlation between the mRNAs obtained by prediction and overall survival (OS) of BC patients. By means of LASSO regression analysis, we set up a survival model. And we used the following formula to calculate the prognostic risk score of patients: Risk score = β_mRNA1_ * exp_mRNA1_ + β_mRNA2_ * exp_mRNA2_ + … + β_mRNAn_ * exp_mRNAn_ (the lower the risk score, the lower the risk of recurrence, where ‘β’ is the regression coefficient of mRNAs obtained by LASSO regression analysis, and ‘exp’ is the expression of corresponding mRNAs). Based on the survivalROC R package, a  receiver operating characteristic (ROC) curve was drawn with risk score against survival status. At the same time, the cut‐off point of risk score was picked by survminer R package which divided patients into high‐ and low‐risk groups. Then, chi‐square test was employed to investigate the correlations between the risk score of the 4‐gene‐based classifier with OS and clinicopathological characteristics.

## 3 RESULTS

### 3.1 Analysis of DEmRNAs of TCGA data

**Figure 2:**
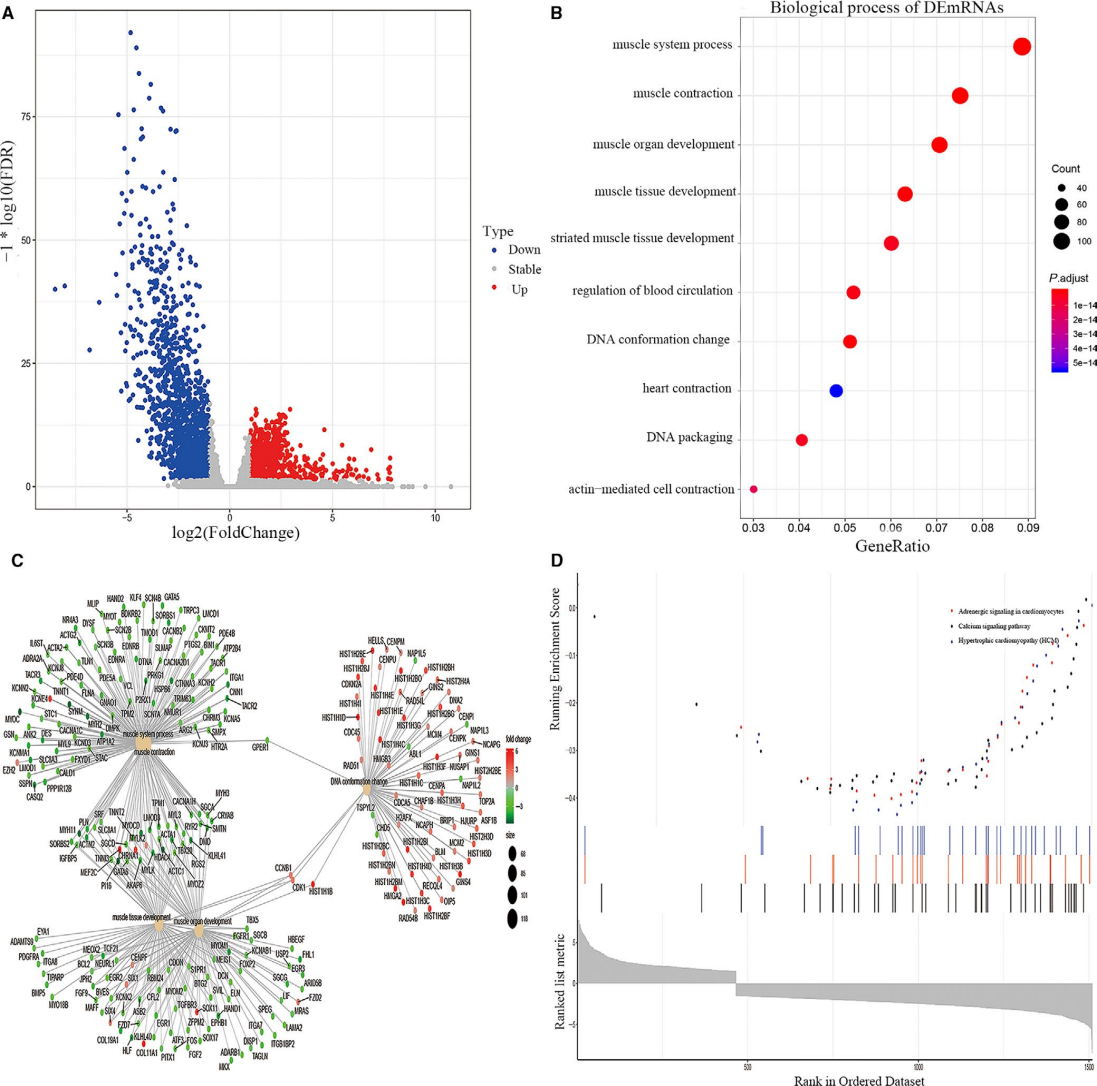
Identification of DEmRNAs from TCGA data. A, Volcano map of DEmRNAs displaying 841 up‐regulated and 1408 down‐regulated mRNAs. B, GO analysis of DEmRNAs showed they may act in muscle system process, muscle contraction and muscle organ development. C, The linkages of DEmRNAs and biological processes. D, KEGG‐GSEA pathway enrichment of DEmRNAs

We identified the DEmRNAs in the TCGA database of BC which contained 841 up‐regulated and 1408 down‐regulated mRNAs. The distribution of the DEmRNAs is shown in Figure 2A. To explore the function of DEmRNAs and their molecular mechanisms in cell cycle progression, we performed GO enrichment analysis on DEmRNAs. GO analysis results showed that DEmRNAs were enriched in muscle system process, muscle contraction and muscle organ development in BP (Figure 2B). Figure 2C depicts the linkages of DEmRNAs and biological terms as a network. In addition, KEGG‐GSEA was used to visualize the distribution of the gene set and the enrichment score. Figure 2D shows DEmRNAs might act through adrenergic signalling in cardiomyocytes, calcium signalling pathway and hypertrophic cardiomyopathy in the development of BC.

### 3.2 Identification of key modules by WGCNA

**Figure 3:**
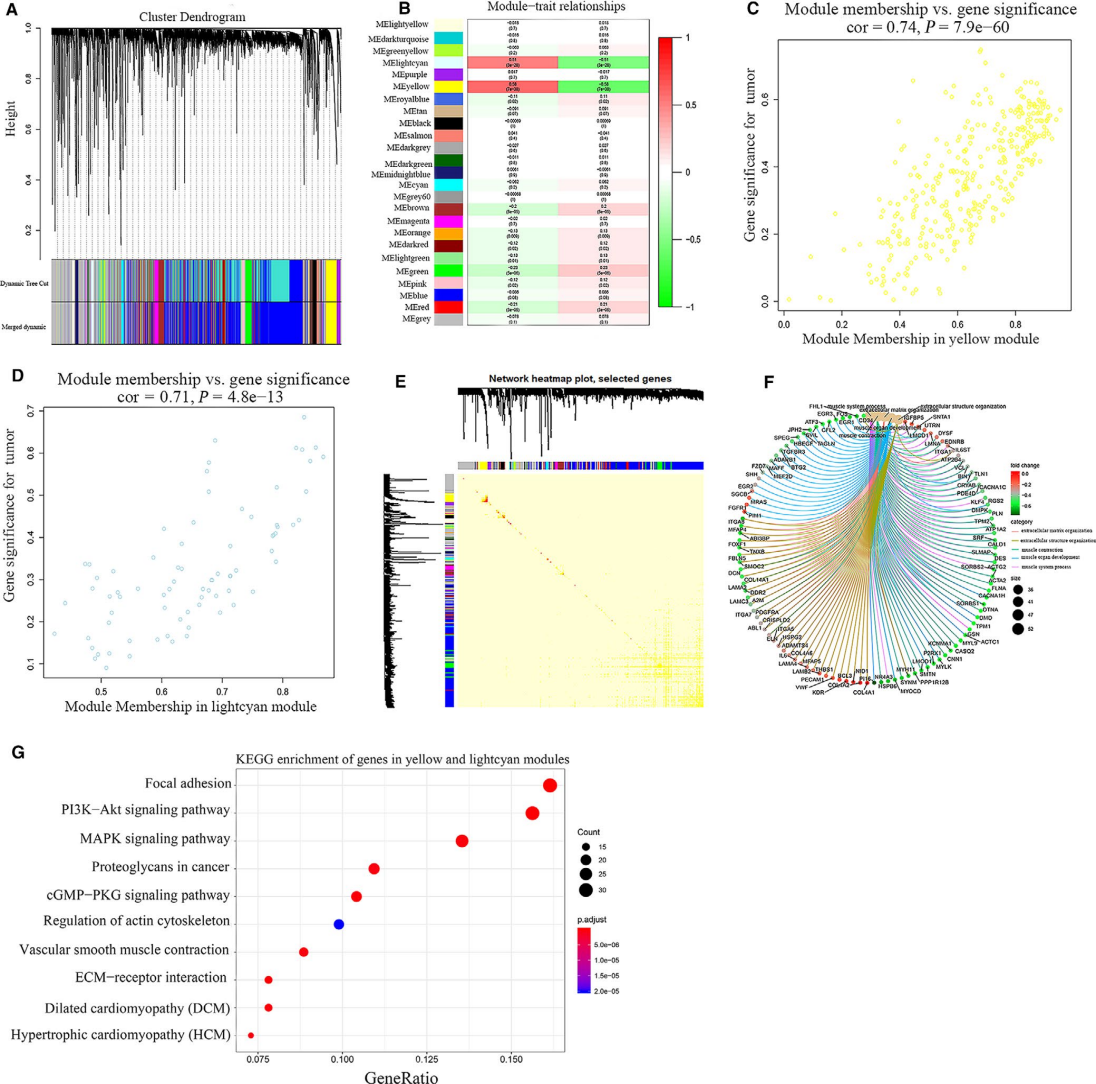
Analysis of key modules highly related to traits. A, Cluster dendrogram of genes with top 60% variances in the co‐expression network. B, The correlation between modules and traits was displayed, and the ME of the yellow and lightcyan modules are most positively related to traits. C‐D, The correlation between GS and MM in the lightcyan and yellow modules. E, TOM plot of selected genes. Modules are sorted by cluster dendrogram. Red colour represents high topological overlap and light colour represents lower overlap. This TOM plot showed that there is no high topological overlap among 25 modules, indicating that the modules have a high degree of scale independence. F, GO‐GSEA showed the linkages of genes and BP. G, KEGG showed the pathway enrichment of genes in yellow and lightcyan modules

Then, we used WGCNA to identify gene co‐expression network modules. After removing unqualified genes and outlying samples, a total of 385 BC and 17 normal samples with 7836 genes (top 60% variances) were left for subsequent analysis. Soft power 8 was picked as the soft thresholding to set up a weighted adjacency matrix (scale‐free R^2^ = 0.86). After the co‐expressed genes were included in a module and the highly correlated modules were merged (the relevance of modules > 0.75), 25 modules were generated (Figure 3A). Among the 25 modules, the module eigengenes (ME) of the yellow and lightcyan modules are most positively related to traits (BC and normal) (Figure 3B). So, the yellow and lightcyan modules were considered as the key module because of high correlation with trait. Figure 3C‐D shows the correlation between gene significance (GS) and module membership (MM) in the lightcyan and yellow modules. Besides, the heatmap plot of selected genes is shown in Figure 3E.

As shown in Figure 3F, we performed GO‐GSEA on the genes in yellow and lightcyan modules to explore their functions in BP. The genes in yellow and lightcyan modules were most related to extracellular matrix and structure organization, and muscle contraction. In addition, genes were highly enriched in focal adhesion, PI3K‐Akt and MAPK signalling pathway, and proteoglycans in cancer by KEGG analysis (Figure 3G), indicating that they might be involved in the process of BC.

### 3.3 Construction of a lncRNA co‐expression network

**Figure 4:**
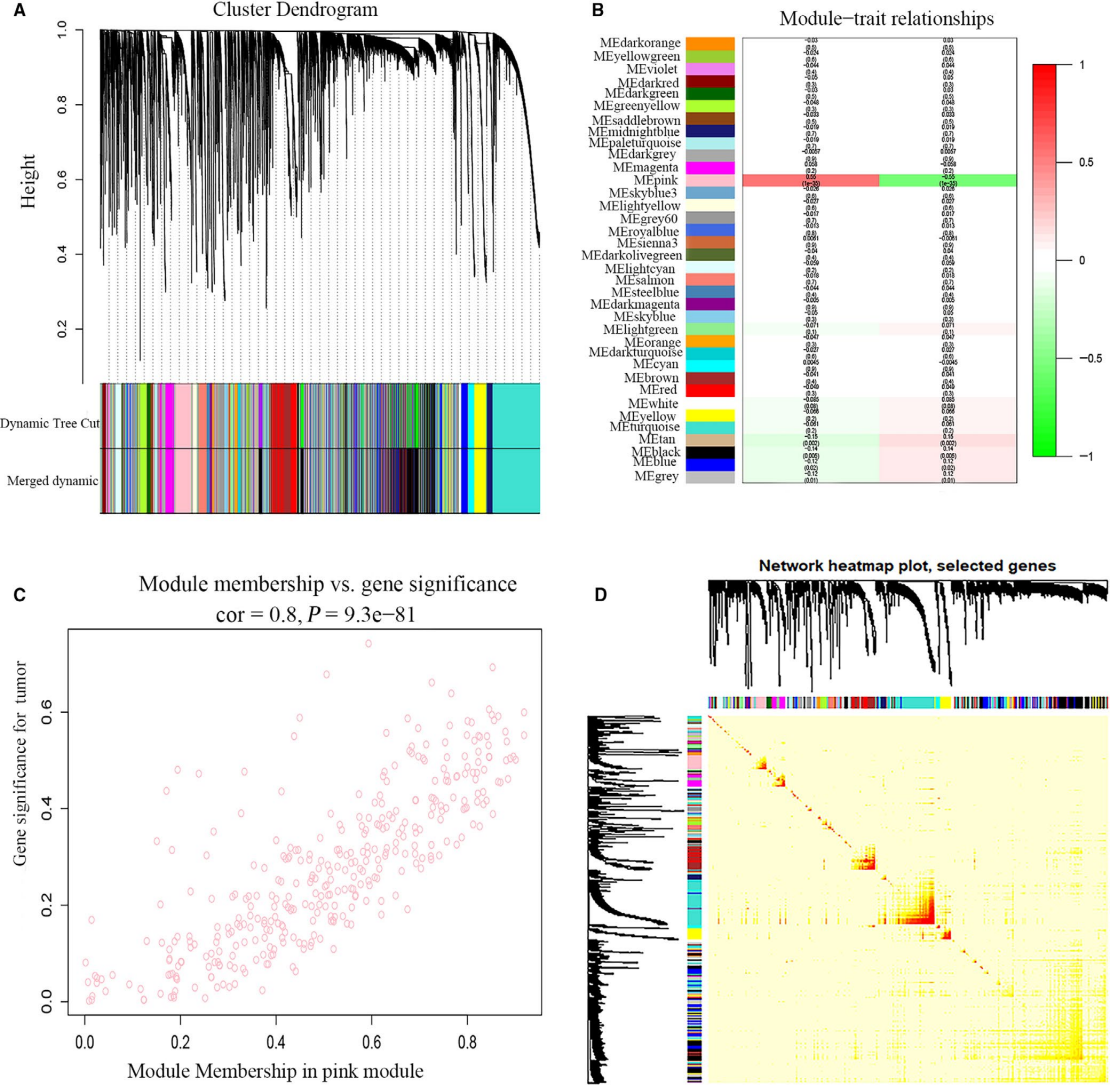
Identification of lncRNAs modules highly related to traits. A, Cluster dendrogram of lncRNAs with top 60% variances in the co‐expression network. B, The correlation between modules and traits were displayed. C, The correlation between GS and MM in the pink module. D, TOM plot of selected lncRNAs

Then, the construction of a lncRNA co‐expression network was aimed to identify the key modules. The top 60% variance lncRNAs (8604 lncRNAs) were selected for WGCNA. Soft power 4 was selected as the soft thresholding to set up a weighted adjacency matrix (scale‐free *R* ^2^ = .89). And a total of 33 modules were generated (Figure 4A). Furthermore, Figure 4B‐C shows that pink module which contained 357 lncRNAs was regarded as key module based on correlation analysis. The network heatmap is shown in Figure 4D.

### 3.4 Overlapped mRNAs of predicted mRNAs, WGCNA mRNAs and DEmRNAs

**Figure 5:**
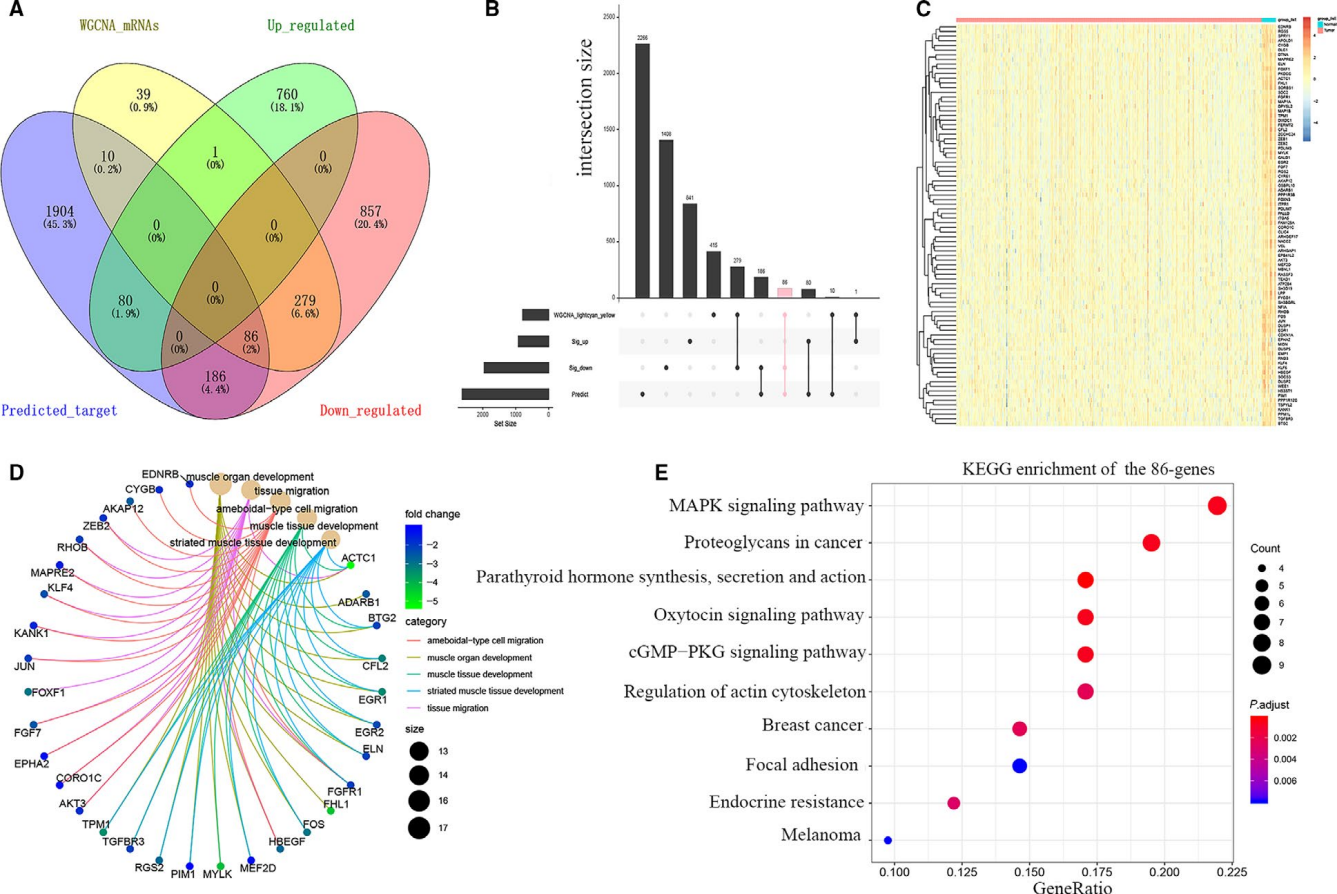
86 mRNAs were displayed by GO and KEGG. A‐B, Common 86 mRNAs among the predicted mRNAs, differentially expressed mRNAs and the mRNAs in the yellow and lightcyan modules. C, The expression heatmap showed the 86 genes were down‐regulated in the patients with tumour. D, The linkages of 86 mRNAs and biological terms in BP were shown. E, KEGG analysis showed the pathway enrichment of 86 mRNAs

By construction of lncRNA co‐expression network, we obtained 357 lncRNAs in the pink module. Then, by miRcode online tool, the 357 lncRNAs were used to predict miRNAs which lncRNAs can bind to them through MREs. At the same time, we obtained the top 400 miRNAs with the highest expression through TCGA miRNA‐Seq. After that, we selected the overlapped miRNAs (55) among the predicted miRNAs (208) and the highest expressed miRNAs (400). And by the means of miRDB, TargetScan, miRTarBase and the starBase dataset, we took advantage of these 55 overlapped miRNAs for the second prediction and obtained 2266 mRNAs. Figure 5A‐B shows that we obtained 86 overlapped mRNAs by analysing the 2266 predicted mRNAs, 2,249 differentially expressed mRNAs (841 up‐regulated and 1408 down‐regulated mRNAs) and the 415 mRNAs in the yellow and lightcyan modules. The expression of 86 overlapped mRNAs, to our surprise, was all down‐regulated. Figure 5C shows the expression heatmap of the 86 genes in 433 samples. The GO analysis was further used to display biological function of 86 mRNAs. Figure 5D shows that the mRNAs were mostly related to ameboidal‐type cell migration, muscle organ development and muscle tissue development. In addition, genes were highly enriched in MAPK signalling pathway, proteoglycans in cancer, parathyroid hormone synthesis, secretion and action by KEGG analysis (Figure 5E).

### 3.5 Construction of the 4‐gene‐based prognostic model

**Figure 6:**
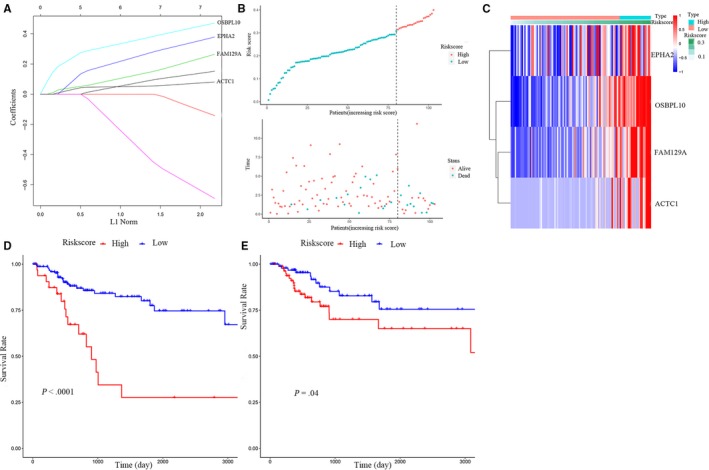
Construction of the 4‐gene‐based prognostic model. A, LASSO coefficient profiles of the 4 survival‐related RNAs. B, BC patients were divided into high‐risk and low‐risk groups. C, The expression heatmap of the 4 genes between high‐risk and low‐risk group. D‐E, Kaplan‐Meier survival curve of OS and DFS in the whole set

A total of 186 patients from TCGA were randomly divided into training set (n = 103) and internal testing set (n = 83) separately. Demographic and clinical data for the training and internal testing set are summarized in Table 1. As shown in Table 1, there is no significant difference in the clinicopathological characteristics. Then, we conducted univariate regression analysis for the 86 genes to explore the genes relevant to the OS of BC patients from the training set; then, a 7‐gene‐based model was constructed with the cut‐off for relevance set at *P* < .05. All the seven genes were then performed with the LASSO regression, and a prognostic model for 1,3,5 years of OS with 4 genes was constructed: ACTC1 + FAM129A + OSBPL10 + EPHA2 (Table 2). Figure 6A shows LASSO coefficient of 4‐gene. After the risk score was calculated for each BC patient, according to the risk scores, we classified the patients into the high‐risk group (n = 23) and low‐risk group (n = 80) with the cut‐off for risk score set at ‘0.3073’. (Figure 6B). Besides, Figure 6C shows the heatmap of the 4 genes expression between the high‐risk and low‐risk group. Kaplan‐Meier survival curves of OS and DFS in the whole sets (n = 186) showed that compared to the low‐risk group, the high‐risk group was tended to lower OS survival rate (*P* < .001), and DFS survival rate (*P* = .04) (Figure 6D,E).

**Table 1:** Clinical features for the BC patients in the training set, testing set

Parameters	Training set n = 103	Testing set n = 83	*χ* ^2^	*P* value
Age				
≤65	41	43	2.2105	.1371
>65	62	40		
Gender				
Male	80	59	0.73567	.3911
Female	23	24		
Subtype				
Papillary	48	35	0.19702	.6571
Non‐Papillary	54	47		
Grade				
Low	12	7	0.20189	.6532
High	91	75		
T stage				
T1‐2	62	48	0.065705	.7977
T3‐4	40	35		
TNM stage				
I‐II	56	39	0.4756	.4904
III‐IV	47	42		
Survival status				
Alive	80	68	0.28411	.594
Dead	23	15		

**Table 2:** Univariate Cox proportional hazard regression analysis and LASSO of 7 genes

Gene	Univariate cox regression analysis			LASSO coefficient
	HR (95%CI)	95% CI	*P* value	
ACTC1	2.766	1.145‐6.679	.024	0.025202
EPHA2	2.388	1.042‐5.477	.040	0.118639
OSBPL10	2.442	1.012‐5.891	.047	0.271164
FAM129A	2.560	1.087‐6.025	.031	0.114802
EGR1	3.226	1.332‐7.814	.010	
PALLD	2.602	1.074‐6.301	.034	

### 3.6 Validation of survival prediction in the integrated test sets

**Figure 7:**
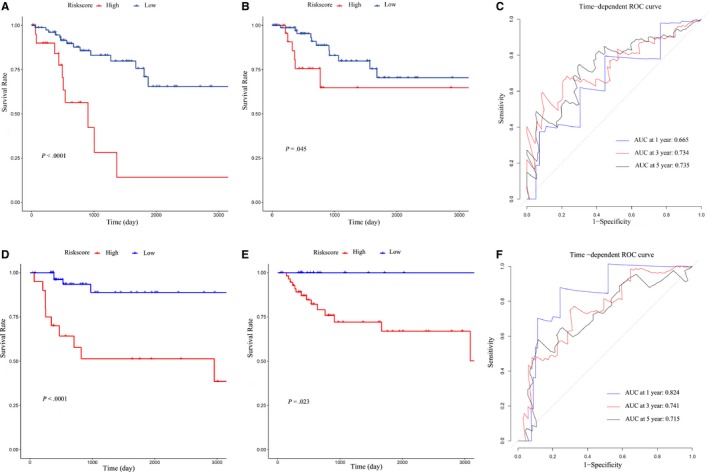
Validation of survival prediction. A‐B, Kaplan‐Meier survival curve of OS and DFS in the training set. C, Time‐dependent ROC curves in the training set. D‐E, Kaplan‐Meier survival curve of OS and DFS in the integrated test set. F, Time‐dependent ROC curves in the integrated test set

To validate the ability of this prognostic model to predict survival of BC patients, we draw Kaplan‐Meier survival curves of OS and DFS in the training and integrated test sets. In the training set, compared to the low‐risk group, the high‐risk group was tended to lower OS survival rate (*P* < .001) and DFS survival rate (*P* = .045) (Figure 7A,B). ROC curve was conducted to analyse the false‐positive rate and true‐positive rate of the 4‐gene‐based prognostic model, and the area under ROC curve based on OS at 1,3,5 years was 0.665, 0.734 and 0.735 (Figure 7C). In addition, we did the same analyses in the internal testing set (Figure 7D,E,F). By the validation, we showed the high accuracy prediction ability of this prognostic model.

### 3.7 Correlation between 4 gene classifier and clinicopathologic characteristics in the training set

Based on TCGA data, we obtained the clinical information of 186 BC patients. Chi‐square test was conducted on 6 clinical factors between the high‐risk and low‐risk group. As shown in Table 3, the 2 clinical characteristics (T stage and TNM stage) showed significant differences between the two groups. The patients in the high‐risk group tended to have higher staging.

**Table 3:** Correlations between risk score of the 4‐gene‐based classifier with OS and clinicopathological characteristics in the training set

Parameters	High risk	Low risk	*χ* ^2^	*P* Value
Age				
≤65	6	35	1.6472	.1993
>65	17	45		
Gender				
Male	14	66	3.6526	.05598
Female	9	14		
Subtype				
Papillary	7	41	1.8934	.1688
Non‐Papillary	15	39		
Grade				
Low	1	11	0.2877169	.2911
High	22	69		
T stage				
T1‐2	9	53	4.7274	.02969
T3‐4	14	26		
TNM stage				
I‐II	7	49	5.6519	.01744
III‐IV	16	31		

### 3.8 Construction of ceRNA network

**Figure 8:**
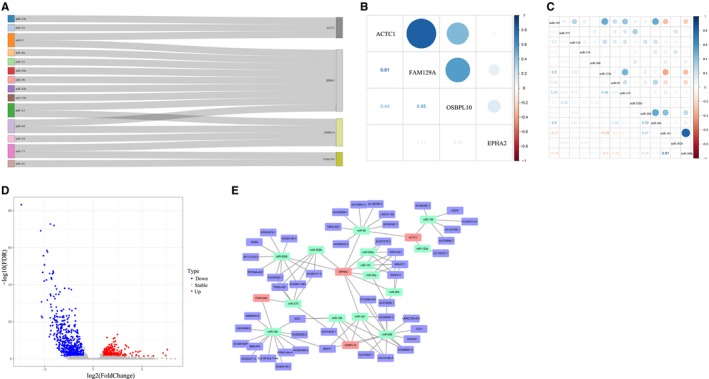
Construction of ceRNA network. A, The linkage among the 4 mRNAs and the 14 miRNAs. B‐C, The correlation of the mRNAs and miRNAs. D, Volcano map of DElncRNAs. E, The construction of a ceRNA network including 4 mRNAs, 14 miRNAs and 48 lncRNAs. The purple spots represent the lncRNAs. The light green spots represent the miRNAs. The pink spots represent the mRNAs

First, the relationship among the 4 mRNAs and the 14 miRNAs which might target the 4 mRNAs were displayed. Among the network, miR‐124 could target OSBPL10 and EPHA2, while EPHA2 was simultaneously regulated by miR‐124, miR‐141, miR‐200a, miR‐26a, miR‐26b, miR‐302b, miR‐506, miR‐520d and miR‐95(Figure 8A). Then, we showed the correlation of mRNAs and miRNAs in Figure 8B‐C. By means of edgeR, the differentially expressed lncRNAs were analysed based on lncRNA expression data from TCGA. A total of 788 differentially expressed lncRNAs (249 up‐regulated and 539 down‐regulated) were identified (Figure 8D). Next, 48 overlapped lncRNAs were obtained between the 788 differentially expressed lncRNAs and the 649 lncRNAs predicted from 14 miRNAs. Finally, Figure 8E shows that we constructed a lncRNA‐miRNA‐mRNA ceRNA network including 4 mRNAs, 14 miRNAs and 48 lncRNAs.

## 4 DISCUSSION

BC, one of the leading causes of cancer death worldwide, is rarely diagnosed in individuals aged < 40 years. But the patient remains at risk for developing a new lesion probably with a more advanced stage when a tumour is diagnosed anywhere within the urothelial tract.^17^ Not surprisingly, after diagnosis of BC, most patients were reported significant decline in health‐related quality of life scores over time.^18^ The histological grade of malignant tumours shows the tumour development and is often used to evaluate tumour differentiation and patients’ prognosis, as well as bladder cancer. But, given the difficulty in obtaining the histological samples of BC patients, we want to explore some novel markers to screen BC earlier and predict the prognosis of patients with BC.

In recent years, increasing ceRNA networks, including mRNAs and lncRNAs, are involved in the process of diseases, especially human tumours. Molecular interaction in ceRNA network could promote co‐ordination of BP, and if the balance is compromised, it can lead to cancer. As a miRNA sponge, lncRNA HULC may be related to tumorigenesis. Once HULC is up‐regulated, it absorbs miR‐372 to the putative binding site and PRKACB is released from the binding of the miR‐372, while PRKACB could phosphorylate CREB, and thus controls HULC transcription 5.^19^ What's more, lncRNA‐miRNA‐mRNA ceRNA network might play a role for prediction prognosis of patients. A study, based on TCGA data, constructed a ceRNA network including 148 mRNAs, 60 miRNAs and 37 lncRNAs. Then by means of multivariate logistic regression, they established a 5‐lncRNA‐based survival model to predict the prognosis of patients with colon adenocarcinoma.^20^ In the hepatocellular carcinoma (HCC), BCYRN1 might regulate some cancer‐related pathways to promote HCC initiation via an lncRNA‐miRNA‐mRNA network. Through combing plasma BCYRN1 with alpha‐fetoprotein, the diagnosis of HCC was remarkably improved.^21^ Therefore, the study of RNA interactions at the molecular level will lead to a better understanding of the gene regulatory network in cancer.

In the present study, EdgeR was conducted to identify the DEmRNAs between the BC and normal bladder samples. Further study, we obtained 86 overlapped mRNAs among the predicted mRNAs, the DEmRNAs and the mRNAs in the yellow and lightcyan modules. And GO analysis showed that the 86 mRNAs were involved in the amoeboid cell migration that was accomplished by extension and retraction of a pseudopodium. Considering that some tumour cell lines have shown inherent amoeboid appearance,^22^ the ameboidal‐type cell migration in which 86 mRNAs participate may be the true mechanism of bladder cancer cell migration. Then, a survival model with 4 genes was constructed by univariate Cox regression and LASSO regression analysis: ACTC1 + FAM129A + OSBPL10 + EPHA2. Based on this model, we predicted risk score of BC patients and identified the high accuracy prediction ability of this prognostic model. At last, 4 mRNAs, 14 miRNAs and 48 lncRNAs were used to construct a ceRNA network. In this ceRNA network, we found that the patients in the high‐risk group tended to have higher staging. Molecularly, mRNA expression is regulated by miRNA and lncRNA. Based on this, miRNAs and lncRNAs might regulate mRNAs and thus affect the biological process and staging of BC.

EphA2, a member of the family of Ephrin receptor tyrosine kinases, is a functional signalling receptor for progranulin. Interaction of progranulin with EphA2 may act in cancer progression and tumour angiogenesis.^23^ Angiogenesis plays an important role in BC. A study found the immunosuppressive drug leflunomide inhibited angiogenesis in bladder carcinogenesis model via significant inhibition of the sEphrin‐A1/EphA2 system and might be a potential biomarker for treating BC beyond immunosuppressive therapy.^24^ In addition, EphA2 had been demonstrated to be essential in ceRNA network. In gastric cancer, miR‐302b exhibited anti‐tumour activity by reversing EphA2 regulation. And this modulation of EphA2 also had distinct effects on cell proliferation and migration in GC.^25^ Besides, ephA2/ephirnA1 signalling regulates miR‐302b expression and attenuates malignant pleural mesothelioma cell growth.^26^ Further study had shown that MIAT/miR‐520d‐3p/ EphA2 might be a new target for HCC therapy. Other studies showed the interaction between miR‐520d and EphA2 could enhance tumour suppression in ovarian cancer and gastric cancer.^27^, ^28^

MiR‐26a and miR‐26b play important roles in BC as tumour suppressors. Overexpression of miR‐26a/miR‐26b inhibited the proliferation and migration of BC cells.^29^, ^30^ Importantly, a study found that blood miR‐26b detected the presence of invasive bladder tumours with 94% specificity and 65% sensitivity.^31^ And with increasing tumour‐nodes‐metastasis staging in bladder cancer, miR‐26b showed a moderate decreasing trend.^32^ As for interaction between EphA2 and miR‐26b in cancers, EphA2 was verified as the target of miR‐26b. MiR‐26b could enhance radiosensitivity of hepatocellular carcinoma cells by targeting EphA2.^33^, ^34^ And overexpression of miR‐26b dramatically inhibited the proliferation, invasion and migration of HCC cells by targeting EphA2.^35^ So, among the network we constructed, we wonder if the interaction between EphA2 and miR‐26b could play roles in tumour angiogenesis and proliferation in BC. We will do further research to validation our hypothesis.

Besides, FAM129A/miR‐195, ACTC1, XIST/ miR‐124/miR‐133a, miR‐506, miR‐200a and miR‐141 were all related to the development of BC.^36^, ^37^, ^38^, ^39^, ^40^, ^41^ Thus, a lot of previous studies have demonstrated the molecular functions of the ceRNA network in the occurrence and development of BC. Among the ceRNA network we constructed, we took advantage of the 4 genes to explore the prognosis of BC patients which indicated the higher risk group was tended to a poor prognosis. And based on the 4 genes, we constructed a ceRNA network as a biomarker for BC patients. However, we do not know how mRNAs, miRNAs and lncRNAs interact in this network. For example, we predicted that lncRNAs could bind miRNAs through MRE and indirectly regulate mRNAs expression. However, as for the specific signalling pathway in which this regulatory network influences specific biological processes, we still do not know which needs further research.

Inevitably, our research had some innate limitations which need to be addressed. Firstly, although this bioinformatics study was designed well, and a strict threshold for WGCNA analysis was set up, the major drawback of our study was the lack of in vivo and vitro validation. Secondly, the current study was of a retrospective nature, as it was based on data from TCGA dataset without validating it in a prospective clinical trial. Despite these drawbacks, the results of our study demonstrate that our survival model with 4 genes could be used as reliable prognostic predictors of BC, as well as the ceRNA network plays a role in the molecular regulatory network of BC.

## CONFLICTS OF INTEREST

The authors declare that there are no conflicts of interest.

## AUTHOR CONTRIBUTIONS

S. L. conceived and designed the study, J. J., Y. B. and X. L. performed the analysis procedures, D. Y., X. Y., J. Y, T. L. and J. J. analysed the results, T. L., and S. L. contributed analysis tools, J. J., and S. L. contributed to the writing of the manuscript. All authors reviewed the manuscript.

## Data Availability

The data generated and analysed during the current study are available from the corresponding author on reasonable request. Public data and data repositories are referenced within the manuscript.

## References

[1] Ebrahimi H , Amini E , Pishgar F , et al. Global, Regional and National Burden of Bladder Cancer, 1990 to 2016: Results from the GBD Study 2016. J Urol. 2019;201:893‐901. 30676477 10.1097/JU.0000000000000025

[2] Grayson M . Bladder cancer. Nature. 2017;551:S33. 29117156 10.1038/551S33a

[3] Pietzak EJ , Bagrodia A , Cha EK , et al. Next‐generation Sequencing of Nonmuscle Invasive Bladder Cancer Reveals Potential Biomarkers and Rational Therapeutic Targets. Eur Urol. 2017;72:952‐959. 28583311 10.1016/j.eururo.2017.05.032 PMC6007852

[4] Kouhsar M , Azimzadeh Jamalkandi S , Moeini A , et al. Detection of novel biomarkers for early detection of Non‐Muscle‐Invasive Bladder Cancer using Competing Endogenous RNA network analysis. Sci Rep. 2019;9:8434. 31182759 10.1038/s41598-019-44944-3 PMC6557814

[5] Chen JB , Zhu YW , Guo X , et al. Microarray expression profiles analysis revealed lncRNA OXCT1‐AS1 promoted bladder cancer cell aggressiveness via miR‐455‐5p/JAK1 signaling. J Cell Physiol. 2019;234:13592‐13601. 30609030 10.1002/jcp.28037 PMC13483269

[6] Salmena L , Poliseno L , Tay Y , et al. A ceRNA hypothesis: the Rosetta Stone of a hidden RNA language? Cell. 2011;146:353‐358. 21802130 10.1016/j.cell.2011.07.014 PMC3235919

[7] Karreth FA , Pandolfi PP . ceRNA cross‐talk in cancer: When ce‐bling Rivalries Go Awry. Cancer Discov. 2013;3(10):1113‐1121. 24072616 10.1158/2159-8290.CD-13-0202 PMC3801300

[8] Qi X , Zhang DH , Wu N , et al. ceRNA in cancer: possible functions and clinical implications. J Med Genet. 2015;52:710‐718. 26358722 10.1136/jmedgenet-2015-103334

[9] Frankish A , Diekhans M , Ferreira AM , et al. GENCODE reference annotation for the human and mouse genomes. Nucleic Acids Res. 2019;47:D766‐D773. 30357393 10.1093/nar/gky955 PMC6323946

[10] Robinson MD , McCarthy DJ , Smyth GK . edgeR: a Bioconductor package for differential expression analysis of digital gene expression data. Bioinformatics. 2010;26:139‐140. 19910308 10.1093/bioinformatics/btp616 PMC2796818

[11] Ashburner M , Ball CA , Blake JA , et al. Gene Ontology: tool for the unification of biology. Nat Genet. 2000;25:25‐29. 10802651 10.1038/75556 PMC3037419

[12] Kanehisa M , Goto S , Furumichi M , et al. KEGG for representation and analysis of molecular networks involving diseases and drugs. Nucleic Acids Res. 2010;38:D355‐D360. 19880382 10.1093/nar/gkp896 PMC2808910

[13] Subramanian A , Tamayo P , Mootha VK , et al. Gene set enrichment analysis: a knowledge‐based approach for interpreting genome‐wide expression profiles. Proc Natl Acad Sci USA. 2005;102:15545‐15550. 16199517 10.1073/pnas.0506580102 PMC1239896

[14] Yu G , Wang LG , Han Y , et al. clusterProfiler: an R package for comparing biological themes among gene clusters. Omics. 2012;16:284‐287. 22455463 10.1089/omi.2011.0118 PMC3339379

[15] Langfelder P , Horvath S . WGCNA: an R package for weighted correlation network analysis. BMC Bioinformatics. 2008;9:559. 19114008 10.1186/1471-2105-9-559 PMC2631488

[16] Li A , Horvath S . Network module detection: Affinity search technique with the multi‐node topological overlap measure. BMC Res Notes. 2009;2:142. 19619323 10.1186/1756-0500-2-142 PMC2727520

[17] Spiess PE , Agarwal N , Bangs R , et al. Version 5.2017, NCCN Clinical Practice Guidelines in Oncology. J Natl Compr Canc Netw. 2017;15:1240‐1267. 28982750 10.6004/jnccn.2017.0156

[18] Smith AB , Jaeger B , Pinheiro LC , et al. Impact of bladder cancer on health‐related quality of life. BJU Int. 2018;121:549‐557. 28990272 10.1111/bju.14047

[19] Wang J , Liu X , Wu H , et al. CREB up‐regulates long non‐coding RNA, HULC expression through interaction with microRNA‐372 in liver cancer. Nucleic Acids Res. 2010;38:5366‐5383. 20423907 10.1093/nar/gkq285 PMC2938198

[20] Chen F , Li Z , Deng C , et al. Integration analysis for novel lncRNA markers predicting tumor recurrence in human colon adenocarcinoma. J Transl Med. 2019;17:299. 31470869 10.1186/s12967-019-2049-2 PMC6717325

[21] Ming XL , Feng YL , He DD , et al. Role of BCYRN1 in hepatocellular carcinoma pathogenesis by lncRNA‐miRNA‐mRNA network analysis and its diagnostic and prognostic value. Epigenomics. 2019;11:1209‐1231. 31339046 10.2217/epi-2018-0218

[22] Sahai E , Marshall CJ . Differing modes of tumour cell invasion have distinct requirements for Rho/ROCK signalling and extracellular proteolysis. Nat Cell Biol. 2003;5:711‐719. 12844144 10.1038/ncb1019

[23] Neill T , Buraschi S , Goyal A , et al. EphA2 is a functional receptor for the growth factor progranulin. J Cell Biol. 2016;215:687‐703. 27903606 10.1083/jcb.201603079 PMC5146997

[24] Chu ML , Zhang CY . Inhibition of angiogenesis by leflunomide via targeting the soluble ephrin‐A1/EphA2 system in bladder cancer. Sci Rep. 2018;8:13. 29367676 10.1038/s41598-018-19788-y PMC5784165

[25] Huang J , He YJ , McLeod HL , et al. miR‐302b inhibits tumorigenesis by targeting EphA2 via Wnt/beta‐catenin/EMT signaling cascade in gastric cancer. BMC Cancer. 2017;17:12. 29273006 10.1186/s12885-017-3875-3 PMC5741943

[26] Khodayari N , Mohammed KA , Lee H , et al. MicroRNA‐302b targets Mcl‐1 and inhibits cell proliferation and induces apoptosis in malignant pleural mesothelioma cells. Am J Cancer Res. 2016;6:1996‐2009. 27725905 PMC5043109

[27] Li RX , Yuan WJ , Mei WJ , et al. MicroRNA 520d–3p inhibits gastric cancer cell proliferation, migration, and invasion by downregulating EphA2 expression. Mol Cell Biochem. 2014;396:295‐305. 25063221 10.1007/s11010-014-2164-6

[28] Nishimura M , Jung EJ , Shah MY , et al. Therapeutic Synergy between microRNA and siRNA in Ovarian Cancer Treatment. Cancer Discov. 2013;3:1302‐1315. 24002999 10.1158/2159-8290.CD-13-0159 PMC3855315

[29] Miyamoto K , Seki N , Matsushita R , et al. Tumour‐suppressive miRNA‐26a‐5p and miR‐26b‐5p inhibit cell aggressiveness by regulating PLOD2 in bladder cancer. Br J Cancer. 2016;115:354‐363. 27310702 10.1038/bjc.2016.179 PMC4973152

[30] Wu K , Mu XY , Jiang JT , et al. miRNA‐26a‐5p and miR‐26b‐5p inhibit the proliferation of bladder cancer cells by regulating PDCD10. Oncol Rep. 2018;40:3523‐3532. 30272373 10.3892/or.2018.6734

[31] Tolle A , Jung M , Rabenhorst S , et al. Identification of microRNAs in blood and urine as tumour markers for the detection of urinary bladder cancer. Oncol Rep. 2013;30:1949‐1956. 23877086 10.3892/or.2013.2621

[32] Gottardo F , Liu CG , Ferracin M , et al. Micro‐RNA profiling in kidney and bladder cancers. Urol Oncol. 2007;25:387‐392. 17826655 10.1016/j.urolonc.2007.01.019

[33] Jin Q , Li XJ , Cao PG . miR‐26b enhances radiosensitivity of hepatocellular carcinoma cells by targeting EphA2. Iran J Basic Med Sci. 2016;19:851‐857. 27746866 PMC5048120

[34] Jin Q , Li XJ , Cao PG . MicroRNA‐26b enhances the radiosensitivity of hepatocellular carcinoma cells by targeting EphA2. Tohoku J Exp Med. 2016;238:143‐151. 26843134 10.1620/tjem.238.143

[35] Li HS , Sun QL , Han B , et al. MiR‐26b inhibits hepatocellular carcinoma cell proliferation, migration, and invasion by targeting EphA2. Int J Clin Exp Pathol. 2015;8:4782‐4790. 26191168 PMC4503040

[36] Zhu NQ , Hou JY , Wu YH , et al. Integrated analysis of a competing endogenous RNA network reveals key lncRNAs as potential prognostic biomarkers for human bladder cancer. Medicine. 2018;97:12. 10.1097/MD.0000000000011887 PMC6392549 30170380

[37] Hou Y . MiR‐506 inhibits cell proliferation, invasion, migration and epithelial‐to‐mesenchymal transition through targeting RWDD4 in human bladder cancer. Oncol Lett. 2019;17:73‐78. 30655740 10.3892/ol.2018.9594 PMC6313135

[38] Zhou KQ , Yang JR , Li XR , et al. Long non‐coding RNA XIST promotes cell proliferation and migration through targeting miR‐133a in bladder cancer. Exp Ther Med. 2019;18:3475‐3483. 31602223 10.3892/etm.2019.7960 PMC6777290

[39] Yang R , Xu J , Hua X , et al. Overexpressed miR‐200a promotes bladder cancer invasion through direct regulating Dicer/miR‐16/JNK2/MMP‐2 axis. Oncogene. 2020;39(9):1983‐1996. 31772330 10.1038/s41388-019-1120-z PMC7044116

[40] Yin XH , Jin YH , Cao Y , et al. Development of a 21‐miRNA Signature Associated With the Prognosis of Patients With Bladder Cancer. Front Oncol. 2019;9:10. 31448232 10.3389/fonc.2019.00729 PMC6692470

[41] Zaravinos A , Lambrou GI , Boulalas I , et al. Identification of Common Differentially Expressed Genes in Urinary Bladder Cancer. PLoS ONE. 2011;6:28. 10.1371/journal.pone.0018135 PMC3070717 21483740

